# The political economy of adult learning systems

**DOI:** 10.1007/s40955-020-00166-z

**Published:** 2020-07-24

**Authors:** Alexandra Ioannidou, Richard Desjardins

**Affiliations:** 1 German Institute of Adult Education—Leibniz Centre for Lifelong Learning, Bonn, Germany; 2 University of California at Los Angeles, Los Angeles, USA

The influence of culture, history, economic conditions and geopolitical developments on the formation of adult education and training systems as well as their embeddedness in a nation’s education system are well known issues in the adult education community, particularly highlighted in comparative adult education research. Relatively new, though, is the idea that adult education is embedded in characteristic regimes of economic and social institutions, which can be understood in terms of a systematic and emerging political economy of adult learning systems (Rees 2013; Desjardins 2017).

The origins of the term political economy can be traced back to Adam Smith, who is regarded as the father of political economy, and in the work of David Ricardo and Karl Marx. Today, the term has been adopted in various disciplines—from political and social science to psychology, education and environmental sciences—when studying the linkages and interactions between politics and economics and their influence on the formation and organisation of economic and social institutions.

With the increasing scholarly interest from political scientists and economists on the study of education and training, a new research strand focusing on the interrelationships between politics and economics, i.e. the political economy of education and training, has been developed.

The seminal work of Hall and Soskice (2001) on the Varieties of Capitalism (VoC) brought education and training to the frontline of comparative political economy research, as education and training was identified as one of the five institutional spheres of political economies (pp. 25–26). The exploration of the linkages between skills and nation-specific institutions such as labour market, industrial relations as well as welfare state formed consequently one of the most interesting research strands in the comparative study of education and training in contemporary period: the political economy of skill formations regimes.

The political economy of adult learning systems has been developed as an offshoot of the debates on comparative welfare state research and considers contributions from different disciplinary perspectives (political science, sociology, education economics) to understanding the causes and consequences of cross-national diversity in adult learning systems across developed democracies.

The core argument in this research strand is that adult learning systems are embedded in specific economic and social arrangements, “they lie at the intersection of a variety of other systems including a nation’s education and training system, labour market and employment system and other welfare state and social policy measures” (Desjardins 2017, p. 232).

Investigating the role of “institutional complementarities” or “institutional packages” in shaping adult learning systems contributes to the large body of literature triggered by the VoC approach in comparative political economy of skill formation regimes (Mayer and Solga 2008; Busemeyer and Trampusch 2012). Yet, analyses of the impact of institutional settings on adult learning systems build at present a relatively small research strand, which mainly focuses at explaining differences in participation in adult learning (cf. Blossfeld et al. 2014; Roosma and Saar 2016). Very little scholarship deals with exploring variation in adult learning systems on the grounds of existing institutional complementarities and how the latter affect patterns of coordination and outcomes of adult learning systems (Desjardins and Rubenson 2013; Desjardins 2017).

This issue of the *Zeitschrift für Weiterbildungsforschung* focusing on the political economy of adult learning systems aims at furthering this debate and addressing questions dealing with the role of diverse institutions in framing adult learning systems. This includes questions on the impact of institutional structures on participation, of governance modes and policy interventions on outcomes of adult learning systems or issues related to the political economy of educational research.

The topic met high interest and attracted sound scholarly work from all over the world.

*Richard Desjardins* and *Alexandra Ioannidou* introduce in their FOKUS-article “The political economy of adult learning systems: some institutional features that promote adult learning participation” into the interdisciplinary research strand of the political economy of adult learning systems. They review recent multi-disciplinary work and different typologies that have emerged out of the field of comparative economics and comparative politics and show how this work has influenced adult education research. Drawing on quantitative analysis of PIAAC, IALS and other OECD data, they provide empirical evidence on cross-national patterns of organized adult learning. This evidence reveals shortcomings in the explanatory framework of existing typologies from comparative welfare state and comparative politics research, despite their virtues. Desjardins & Ioannidou suggest that there are some specific institutional features that seem to be more proximally related to adult learning systems and appear to play a leading role in explaining the cross-national patterns of variation in the take up, provision and distribution of organised adult learning. Among them: open and flexible formal education structures, public support for education, active labour market policies and programmes that target socially disadvantaged adults.

Explaining variation in participation in adult learning across countries as well as within countries is a challenge for researchers. Improvements with regard to availability and accessibility of international comparable data and advancements in empirical methods enable new insights in this respect. The next two contributions by Miroslav Stefanik and Veronika Philipps deal with explaining cross national participation patterns in adult learning in Europe focusing specifically on the participation of disadvantaged groups.

*Miroslav Stefanik* in his contribution “Multi-layered Perspective on the Barriers to Learning Participation of Disadvantaged Adults” takes a closer look at disadvantaged adults and proposes a multilevel explanatory model that aims to explain the variability behind participation in adult learning. Departing from available theoretical and empirical research on constraints with regard to participation in adult learning, he explores the barriers to participation among disadvantaged adults across Europe. He focuses on employed adults, specifically on three vulnerable sub-groups: low-skilled; young and low-skilled, and immigrants. He uses microdata from the European Union Labour Force Survey (EU-LFS) for 28 European countries and considers also regional variability by applying a multilevel modelling technique. His model groups the explanatory variables into individual-level determinants, household-level, job-related, employer-level related as well as system-level characteristics. Comparing the results across the vulnerable groups and types of determinants yields interesting insights in understanding the variability in adult learning participation across Europe.

*Veronika Philipps* in her contribution “Job-related further education of the elderly in Europe: Do institutions matter?” investigates country differences in the participation in further education for older people from an institutional-theoretical perspective. Departing from the empirical evidence that people in their late employment age are rarely involved in job-related further education in Europe she poses the question whether institutions matter. In line with the literature she argues that institutions function in conjunction with one another and that institutional configurations of the labour market and education system account for the relative disadvantages of older people in participation in further education. She identifies four combinations of factors and examines her hypotheses with Qualitative Comparative Analysis (QCA) drawing on data from the European Union Adult Education Survey (AES) in 26 countries. In contrast to other quantitative methods, the QCA allows for examining not only the influence of isolated factors but also the effect of combinations of institutions. The results of her analysis illustrate how important institutional configurations are for the explanation of disparities in further education. In particular, the existence of far-reaching state and well-developed company structures concerning the provision of continuing education as well as a dual vocational training system are crucial for the appearance of relatively lower disadvantages for older people in a given country.

The next two contributions deal with policy changes and educational reforms in adult learning systems in three different countries: Switzerland, Canada and Aotearoa New Zealand.

*Michael Geiss* reconstructs in his contribution “In Steady Search for Optimization: The Role of Public and Private Actors in Switzerland’s Political Economy of Adult Education” the changes in the political economy of adult education in Switzerland since the middle of the 20th century. He argues that, contrary to the literature, the Swiss adult education system is neither exclusively a market-led nor a stakeholder-led regime and deals with the contradictory accounts of the Swiss adult education system. He shows that alongside with the ongoing power of the employers, industry and professional associations, and the liberal-minded national authorities in Switzerland, there are several other corporate actors that played an important role in recent developments in the Swiss adult education system. The article underlines that “history matters” and that the current situation cannot be understood without considering the several government initiatives since the 1970s as well as the efforts of the private national continuing education association SVEB. It concludes with reflections on the expected impact of the first national law on continuing education, which was enacted in 2017. Methodologically, the analysis is based on historical institutionalism and concentrates on path dependencies and critical junctures.

*Judith Walker* examines in her contribution “Comparing adult education ‘systems’: Canada and Aotearoa New Zealand” recent policy initiatives in adult education and training in the two countries in relation to previous political and educational reforms, taking an explicit comparative perspective. Both Canada and Aotearoa New Zealand are liberal market economies (LME) with high standards of living, high levels of education, and long histories in the evolution of non-formal adult education. The reforms in both countries share many similarities with their emphasis in skill development, increasing immigration and cultural diversity, and a need to address the legacy of colonisation and marginalisation of indigenous populations. Yet, Walker assigns a dearth of adult education infrastructure in Canada as well as insufficient coordination across the institutions due to a fragmented fiscal federalism. In contrast, New Zealand’s centralised policy making, extreme neoliberal reforms of the 1980s and 1990s and subsequent reaction to them during the following decades, resulted in the creation of structures and institutions that allowed for a highly coordinated, regulated, professionalised, and centralised adult education system. Walker’s contribution is in the tradition of comparative education research, which takes the nation state as unit of analysis and shows how two LMEs prioritise and approach adult education and training in relation to their unique socio-cultural-historical-political contexts. This reveals also in their reaction to the COVID-19 pandemic.

*Michael Schemmann, Dörthe Herbrechter *and *Martina Engels* choose a different approach in their contribution about “Researching the Political Economy of Adult Learning Systems: Theoretical Amendments and Empirical Findings”. Their focus lays with the level of adult education organisations, which is hardly taken into account when analysing the political economy of adult learning systems. They introduce neo-institutional ideas to specify aspects of the institutional environment of adult education organizations and outline how these theoretical amendments complement the political economy approach and the governance perspective. By re-analysing interview data from public adult education organizations in Germany they aim at exploring the potential of their theoretical amendments. Their findings provide first empirical indications that the institutional conditions that shape adult learning systems can be further specified with regard to the adult education organizations and their institutionally embedded interdependency relationships with other (organized) actors in their environment.

Finally, *Shalini Singh* looks at the political economy of adult educational research focusing on the “Impact of International Large-Scale Assessments on Adult Education Systems”. She takes as a starting point the fact that international large-scale assessments (ILSAs) have become a predominant tool for the measurement of learning outcomes and the sustainability of education systems all over the globe, often despite resistance from several stakeholders. She claims that especially after the adoption of the Sustainable Development Goals (SDGs) in 2015 ILSAs influence state policies directly or indirectly, irrespectively of whether states participate in them or not. She discusses the role of ILSAs in shaping education systems in general and adult education systems in particular. Her methodology includes document analysis, comparative analysis of four ILSAs and the indicators developed by the Global Alliance to Monitor Learning (GAML) as well as mapping of policy linkages between ILSAs and employability policies. The selected ILSAs include the Programme for the International Assessment of Adult Competencies (PIAAC) by the OECD, the Skills Towards Employability and Productivity (STEP) Survey by the World Bank Group (WBG), School-to Work Transition Survey (STWS) by the ILO and Literacy Assessment and Monitoring Program (LAMP) by the UNESCO Institute of Statistics (UIS). The paper views the changes in adult education systems as a part of global policy shift led by the OECD with ILSAs being some of the effective catalysts and acting as windows of opportunity for introducing changes in the education systems.

Together, these contributions on the main topic help to highlight that theory building and comparative empirical evidence are indispensable for furthering the development of this research strand. We hope that we were able to contribute to these efforts with this issue and would like to thank the editors and the editorial team of the ZfW for their support.

In the rubric FORUM, this issue presents two articles. In their contribution “Assessing media pedagogical competence of adult education teachers”, *Bernhard Schmidt-Hertha* and *Matthias Rohs *address the (digital) media competence of adult educators, a topic that acquired an increasing importance due to the corona pandemic. It is surprising that despite the intensive academic discussion on the topic, it remains largely unclear what can be understood by ‘media-pedagogical competencies’. This includes the question whether the concept describes a specific competence or rather a cross-sectional competence. To develop a structural model of digital media competence, the authors proceed in two steps: First, they engage with the international theoretical discussion; then, they include the specific practical knowledge of experts in adult education. The structure of the model comprises the following facets: field competence, subject-related media competence, subject-didactic competence, pedagogical competence as well as attitudes and self-monitoring. This model (except for media-related competence in different subjects) is tested by using a sample of more than 600 adult education teachers. The empirical results largely confirm the assumptions of the developed model.

The article “Reversing the Matthew Principle—Adult Education in the context of low numeracy” by *Luise Krejcik* and *Anke Grotlüschen* addresses another topic that has been intensely discussed for many years in educational research generally as well as in adult education research: The so-called Matthew effect that points to the relation between prior educational experience and participation in adult education. This effect has been confirmed by research multiple times. Using a re-analysis of PIAAC-data, the authors examine adults with low numerical skills in order to find out whether this effect may be reverse if one does not take into account only participation, but the volume of participation in adult education. The re-analysis shows that both on an OECD-average and in the majority of the Nordic countries, adults with low numerical skills tend to participate less in further education than adults with high numerical skills. But if they participate, the duration of participation is longer than average, also if sociodemographic and occupational factors are controlled. The authors discuss distinct country-specific differences in the context of different welfare state policies.

## References

[CR1] Blossfeld H-P, Kilpi-Jakonen E, Vono de Vilhena S, Buchholz S (2014). Adult learning in modern societies. An international comparison from a life-course perspective.

[CR2] Busemeyer MR, Trampusch C (2012). The political economy of collective skill formation.

[CR4] Desjardins R (2017). Political economy of adult learning systems. Comparative study of strategies, policies and constraints.

[CR3] Desjardins R, Rubenson K (2013). Participation patterns in adult education: the role of institutions and public policy frameworks in resolving coordination problems. European Journal of Education.

[CR5] Hall PA, Soskice D, Hall PA, Soskice D (2001). An introduction to varieties of capitalism. Varieties of capitalism. The institutional foundations of comparative advantage.

[CR6] Mayer KU, Solga H (2008). Skill formation: interdisciplinary and cross-national perspectives.

[CR7] Rees GM (2013). Comparing adult learning systems. An emerging political economy. European Journal of Education.

[CR8] Roosma E, Saar E (2016). Adults who do not want to participate in learning. A cross-national European analysis of their perceived barriers. International Journal of Lifelong Education.

